# Genetic evidence for the involvement of Dicer-like 2 and 4 as well as Argonaute 2 in the *Nicotiana benthamiana* response against *Pelargonium line pattern virus*

**DOI:** 10.1099/jgv.0.001656

**Published:** 2021-10-08

**Authors:** Miryam Pérez-Cañamás, Elizabeth Hevia, Konstantina Katsarou, Carmen Hernández

**Affiliations:** ^1^​ Instituto de Biología Molecular y Celular de Plantas (Consejo Superior de Investigaciones Científicas-Universidad Politécnica de Valencia). Ciudad Politécnica de la Innovación, Ed. 8E. Camino de Vera s/n, 46022 Valencia, Spain; ^2^​ Institute of Molecular Biology and Biotechnology, Foundation for Research and Technology, GR-7110 Heraklion, Crete, Greece

**Keywords:** AGO2, antiviral RNA silencing, DCL, *Nicotiana benthamiana*, RNA virus, PLPV

## Abstract

In plants, RNA silencing functions as a potent antiviral mechanism. Virus-derived double-stranded RNAs (dsRNAs) trigger this mechanism, being cleaved by Dicer-like (DCL) enzymes into virus small RNAs (vsRNAs). These vsRNAs guide sequence-specific RNA degradation upon their incorporation into an RNA-induced silencing complex (RISC) that contains a slicer of the *Argonaute* (AGO) family. Host RNA dependent-RNA polymerases, particularly RDR6, strengthen antiviral silencing by generating more dsRNA templates from RISC-cleavage products that, in turn, are converted into secondary vsRNAs by DCLs. Previous work showed that *Pelargonium line pattern virus* (PLPV) is a very efficient inducer and target of RNA silencing as PLPV-infected *Nicotiana benthamiana* plants accumulate extraordinarily high amounts of vsRNAs that, strikingly, are independent of RDR6 activity. Several scenarios may explain these observations including a major contribution of dicing versus slicing for defence against PLPV, as the dicing step would not be affected by the RNA silencing suppressor encoded by the virus, a protein that acts via vsRNA sequestration. Taking advantage of the availability of lines of *N. benthamiana* with DCL or AGO2 functions impaired, here we have tried to get further insights into the components of the silencing machinery that are involved in anti-PLPV-silencing. Results have shown that DCL4 and, to lesser extent, DCL2 contribute to restrict viral infection. Interestingly, AGO2 apparently makes even a higher contribution in the defence against PLPV, extending the number of viruses that are affected by this particular slicer. The data support that both dicing and slicing activities participate in the host race against PLPV.

## Introduction

Viruses must evade the defence mechanisms of the host in order to establish a productive infection. In plants, one of the main defence responses against both DNA and RNA viruses is that based on RNA silencing [1]. This response is triggered by double-stranded (ds) RNAs that, in the case of RNA viruses, may correspond to viral replication intermediates (generated by the viral RNA dependent-RNA polymerase, RDR) or to highly structured regions of the viral genome [2]. These dsRNAs are recognized by RNase III type enzymes, called Dicer-like (DCL), that cleave them into primary small RNAs (sRNAs) of 21–24 nt [3, 4]. One strand of the small duplex is incorporated into an RNA-induced silencing complex (RISC) whose effector component is an endonuclease of the Argonaute (AGO) family [5, 6]. The sRNA guides RISC to cognate RNA usually leading to its degradation through AGO-mediated slicing [7]. Host RDRs may participate in the generation of the initial dsRNA triggers and are also involved in the amplification of the silencing process by catalysing sRNA-primed generation of new dsRNAs from RISC-cleaved (or other aberrant) products that, in turn, are converted into secondary sRNAs by DCLs [8]. Besides the involvement of sRNAs in cell autonomous silencing, these small molecules can move both short and long distances and thereby also trigger non-cell autonomous local and systemic silencing [9].

Studies mainly performed with the model organism *Arabidopsis thaliana* have shed light on the particular silencing components that participate in antiviral defence. Among the four DCLs encoded by this plant species (DCL1-4), three of them, DCL4, DCL2 and DCL3, seem to function in a hierarchical fashion to yield viral sRNAs (vsRNAs) of 21, 22 and 24 nt, respectively [10–12]. Concerning AGO and RDR proteins, *A. thaliana* encodes ten distinct AGOs and six different RDRs, with AGO1, AGO2 and RDR6 being particularly important in the fight against viruses [13–16]. The identity of the host components participating in antiviral silencing in other plant species has been comparatively less well explored [17].

In order to survive, viruses have developed strategies to counteract antiviral silencing, prominent among which is the production of proteins, called viral suppressors of RNA silencing (VSRs), that interfere at different stages of the silencing pathway [17]. For instance, a common strategy adopted by many unrelated VSRs consists in sRNA binding, which will impair both RISC loading and amplification of silencing, thus weakening host defence [18–21]. Long dsRNAs may also be targets of VSRs as well as protein components of the silencing machinery [22].

From a theoretical point of view, dicing would be sufficient to clear or diminish virus accumulation in infected cells. Upon onset of an active infection, vsRNAs are both generated and used to target viral RNAs leading, as mentioned above, to their degradation. However, if the infection can be adequately controlled, the necessity to sort viral vsRNAs into RISC might conceivably become less critical. Indeed, dicing, rather than slicing, has been reported to play a main role in the latency of some animal viruses in persistently infected cells [23]. In the case of plant viruses, the mere existence of antiviral RISCs was also a matter of debate for some time [3], though the debate has largely been settled once the first evidences of vsRNA-RISCs were obtained [24, 25]. Nevertheless, the possibility of Dicer-mediated processing of viral dsRNAs being enough to impede multiplication of, at least, some plant viruses cannot be completely ruled out, particularly in the case of viruses with lifestyles that present resemblances with those of persistent viruses [26, 27].

*Pelargonium line pattern virus* (PLPV) is the type member of the new genus *Pelarspovirus* in the family *Tombusviridae* [28]. PLPV possesses a single-stranded positive RNA genome of about 4 kb that encodes two proteins involved in replication (p27 and p87), two small movement proteins (p7 and p9.7) and a protein (p37) that acts as both coat protein (CP) and VSR [29–31]. We have shown that *Nicotiana benthamiana* plants infected by PLPV accumulate the highest percentage of vsRNAs reported so far for a plant virus (90 % of vsRNAs with regard total sRNAs), which illustrates the strong defence that the host deploys against the virus. Such strong antiviral silencing was proposed to be a key factor in determining the low viral titers that characterize PLPV infections that, moreover, use to be asymptomatic [32]. It was also suggested that the elevated amount of vsRNAs could reflect the importance of dicing versus slicing in anti-PLPV host response, particularly taking into account that the PLPV-encoded VSR exerts its action mainly through vsRNA sequestration [31], which would prevent RISC assembly (and thus slicing). Reinforcing this possibility, the amount of vsRNAs detected in PLPV-infected *N. benthamiana* was independent from RDR6 activity, which could be influenced by the absence of AGO-cleaved RNAs that serve as RDR substrates. Here we have pursued to assess the relative contribution of DCL and AGO proteins in anti-PLPV silencing. To this aim, we have used *N. benthamiana* plants with DCL or AGO functions impaired either by downregulation of expression through an RNA silencing (also called RNA interference, RNAi) approach (DCL1-4) or by protein inactivation through a CRISPR/Cas9 strategy (AGO2) [33–35]. Results have shown that DCL4 and, to lesser extent, DCL2 contribute to restrict viral infection. Interestingly, AGO2 was also found to be a key factor in the host defence against PLPV, highlighting the importance of slicing in anti-PLPV silencing on one side, and extending the number of viruses that are affected by this particular slicer on the other. Taken together, the data support that both dicing and slicing activities participate in the host race against PLPV.

## Methods

### Plant material and viral inoculation

Different lines of *N. benthamiana* plants were used for PLPV inoculation: (i) wild-type (wt) *N. benthamiana*, (ii) *N. benthamiana* transgenic plants in which the different DCL genes were downregulated, either individually or in double or triple combinations, by expressing hairpin constructs (homozygous lines DCL1.13i, DCL2.11i, DCL3.10i, DCL4.9i, DCL2/4.5i, and hemizygous line DCL3.10×2/4.5i, that corresponds to the progeny that results from crossing DCL3.10i as a female and DCL2/4.5i as a male) [33, 34] and (iii) *N. benthamiana* transformant line with a CRISPR/Cas9 modified AGO2 allele leading to dysfunctional AGO2 protein [35]. All plants were grown until four-to-six leaf stage under greenhouse conditions (16 h day at 24 °C, 8 h night at 20 °C) and then mock or virus-inoculated (see below).

### Virus inoculation

*N. benthamiana* plants were agroinoculated with binary plasmids containing either a wt PLPV cDNA or a mutated PLPV cDNA (hereinafter PLPV-mutp37) carrying three nucleotide replacements in the p37 gene leading to an aminoacid (aa) substitution (W28A). As described previously [31], such aa substitution affects a GW motif in p37 that is critical for its VSR function but not for its encapsidation function. In the mentioned plasmids, that are based on the binary vector pMOG800 [29], the PLPV cDNA is flanked by the *Cauliflower mosaic virus* 35S promoter and the terminator sequence of the *Solanum tuberosum* proteinase inhibitor II gene. Binary construct for expression of tombusvirus p19 (used for VSR function in some experiments) has been described elsewhere [31]. The plasmids were used to transform *Agrobacterium tumefaciens* strain C58C1 by freeze/thaw shock method. Cultures of *A. tumefaciens* harbouring the corresponding plasmid were infiltrated at an OD_660_ of 0.5 on the abaxial side of *N. benthamiana* leaves (two leaves per plant) using a 20 ml needleless syringe. Usually the whole leave was infiltrated unless stated otherwise. Mock inoculated plants were used as controls. All inoculated plants were kept under greenhouse conditions. Local leaves were collected at 7 days post-inoculation (days p.i.) and systemic leaves at either 34 (for wt virus) or 42 (for mutant virus) days p.i. Plant batches for each experiment included 4–6 plants and experiments were repeated at least three times.

### Analysis of viral infection through Northern blot, dot blot and tissue printing hybridization

Total RNA preparations from *N. benthamiana* leaves were obtained by phenol extraction and lithium precipitation [36]. For Northern blot analysis, 4 µg of total RNAs were denatured by glyoxal-dimethyl sulfoxide treatment, electrophoresed in 1 % agarose gels and blotted to nylon membranes (Hybond N+, GE Healthcare). For dot hybridization, 2 µg of total RNAs were directly applied to nylon membranes. For tissue printing hybridization, stems or leaf blades were directly imprinted onto nylon membranes. After UV-crosslinking, membranes were incubated with a ^32^P-radioactive RNA probe for detection of PLPV RNAs. Such a probe was generated by *in vitro* transcription of a pBluescript KS(+)-based construct containing the PLPV p37 gene (nt 2621 to 3637 of PLPV genome). Hybridization signals were visualized by autoradiography or with a PhosphorImager (Fujifilm FLA-5100).

## Results

### DCL4 and, to lesser extent, DCL2 are the main DCL players in *N. benthamiana* anti-PLPV defense

**Fig. 1.**
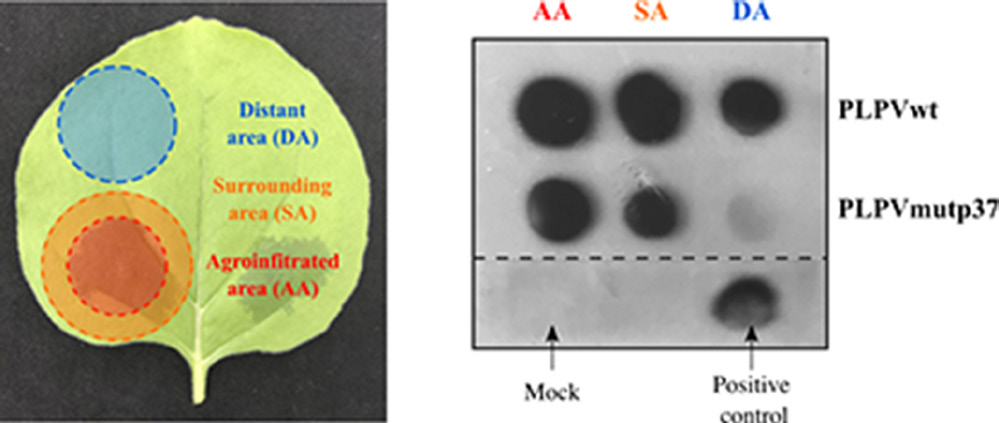
Assessment of local movement of PLPV-p37mut in *N. benthamiana* leaves. PLPV-mutp37 or wt PLPV were agroinfiltrated along with the tombusviral VSR p19 in a small patch of wt *N. benthamiana* leaves (left panel) and the presence of the virus in distinct leaf areas was analysed at 5 days p.i. through dot blot hybridization (right panel). Total RNA from mock inoculated and PLPV-infected plants were used as negative (mock) and positive controls, respectively, for dot blot hybridization as labelled in the right panel. Red: agroinfiltrated area (AA). Orange: surrounding area (SA). Blue: distant area (DA).

**Fig. 2.**
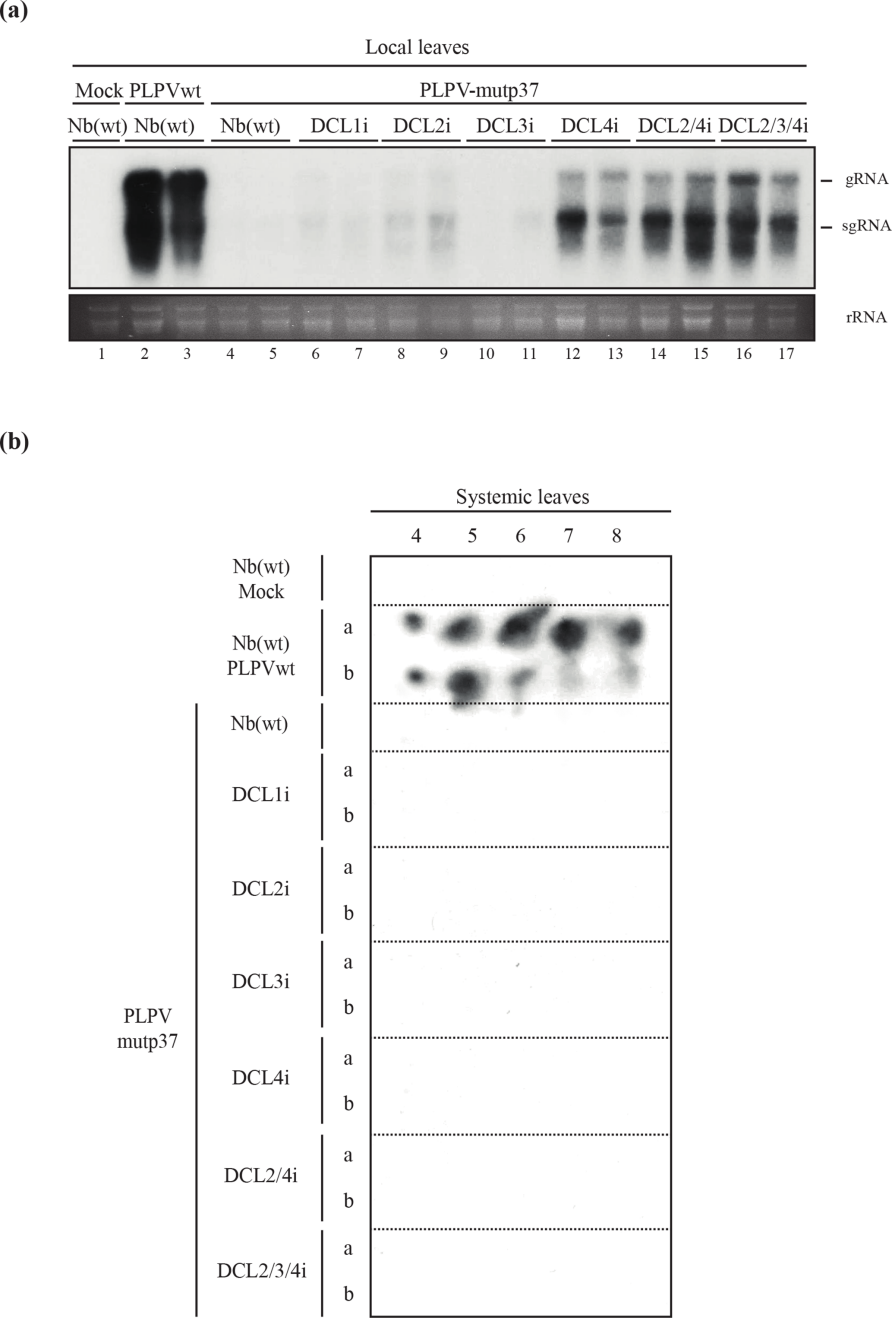
Accumulation of a VSR-deficient PLPV mutant in lines of *N. benthamiana* with downregulated DCL content. PLPV-mutp37 was agroinoculated in wt and in single (DCL1-4), double (DCL2/4) and triple (DCL2/3/4) RNAi lines and local and systemic leaves were harvested at 7 days p.i. and 42 days p.i., respectively, for further analysis. For comparison purposes, wt PLPV was inoculated in parallel on wt *N. benthamiana* plants and local and systemic leaves were collected at the same times. (a) Northern blot analysis of local leaves of different *N. benthamiana* lines (as shown above the lanes) inoculated with either wt PLPV (lanes 2–3) or PLPV-mutp37 (lanes 4–17). Leaves from mock-inoculated plants (lane 1) were used as negative controls. An antisense ^32^P-labelled riboprobe covering the p37 gene was used for hybridization. Positions of the PLPV genomic (g) RNA and of the subgenomic (sg) RNA that the virus produces for expression of 3′-proximal genes, are indicated on the right. Duplicate samples are shown for each virus–plant line combination. Ethidium bromide staining of rRNAs is included below the blots as loading controls. (b) Representative tissue printing hybridization of systemic leaves from mock and PLPV (wt or mutp37)-inoculated plants using a PLPV-specific ^32^P-labelled riboprobe as above. Results from two plants (a, b) are shown for every virus–plant line combination but the outcome was identical for other plants of each series. Plant batches for each experiment included four–six plants and experiments were repeated a minimum of three times.

As *A. thaliana*, *N. benthamiana* encodes four DCL-type RNases [37]. Previous characterization of vsRNAs in PLPV-infected *N. benthamiana* plants showed that those of 21 and 22 nt are the most abundant ones in the vsRNA population with percentages of 41.65 and 32.90 %, respectively, while the fraction of vsRNAs of 24 nt is much lower (0.82 %) as well as those of other size classes [32]. These results suggested a main involvement of DCL4 and DCL2 in anti-PLPV silencing as reported in other plant–virus combinations [11, 38, 39]. In order to confirm, or refute, this possibility, we used a genetic approach by taking advantage of the previous creation of a collection of *N. benthamiana* transgenic lines in which the different DCL genes were downregulated, either individually or in combinations, through an RNAi approach [33, 34, 40]. This collection included either single DCL RNAi lines (henceforth DCL1i, DCL2i, DCL3i or DCL4i) or double (DCL2/4i) and triple (DCL2/3/4i) DCL RNAi combinations. As the presence of an active VSR may mask the effect of silencing components involved in the host defence against a virus [35, 39], we employed a VSR-deficient PLPV mutant, PLPV-mutp37, to study the influence of DCL downregulation on the progression of PLPV infection. This mutant carried the W28A aa replacement in p37 that impaired its VSR activity but preserved its packaging function [31]. Preservation of that function was most likely essential for virus cell-to-cell movement and, indeed, PLPV-mutp37 was perfectly able to spread locally beyond an agroinfiltrated patch when VSR activity was provided *in trans* with another viral suppressor (Fig. 1). Inoculation of wt *N. benthamiana* plants with this PLPV mutant and analysis of viral infection by Northern blot hybridization showed that viral accumulation was barely detectable at 7 days p.i. in local leaves, in agreement with previous results [31] (Fig. 2a, lanes 4–5). Consistently with this deficient local infection, analysis of the inoculated plants at 42 days p.i. revealed that the mutant virus was unable to become systemic (Fig. 2b).

In contrast with the results obtained in wt *N. benthamiana* plants, local leaves of several DCL RNAi lines inoculated in parallel with PLPV-mutp37 exhibited from moderate to elevated virus accumulation levels. In single DCL RNAi lines, the higher increase was observed in the DCL4i line, whereas a modest increase was recorded in the DCL2i line (Fig. 2a, lanes 13–14 and 8–9, respectively). PLPV-mutp37 accumulation in DCL1i or DCL3i lines (Fig. 2b, lanes 6–7 and 10–11, respectively) was very low and comparable to that detected in wt *N. benthamiana* (lanes 4–5) (Fig. 2a). The augmentation of PLPV-mutp37 titers was also remarkable in double DCL2/4 and triple DCL2/3/4 RNAi lines (Fig. 2a, lanes 14–17); indeed, it was frequently higher than that recorded in DCL4i or DCL2i lines (Fig. 2a and data not shown), pointing to an additive effect of the DCLs involved in anti-PLPV silencing. Nevertheless, titers of PLPV-mutp37 in local leaves of single, double or triple DCL RNAi lines were below those reached by wt PLPV in local leaves of wt *N. benthamiana* (Fig. 2a, lanes 2–3), indicating that downregulation of DCLs may compensate, though not totally, the absence of VSR activity in the virus. In consonance with this view, the VSR-deficient PLPV was not detected in systemic leaves of any of the DCL RNAi lines, while the wt virus became systemic in all inoculated wt *N. benthamiana* plants (Fig. 2b).

**Fig. 3.**
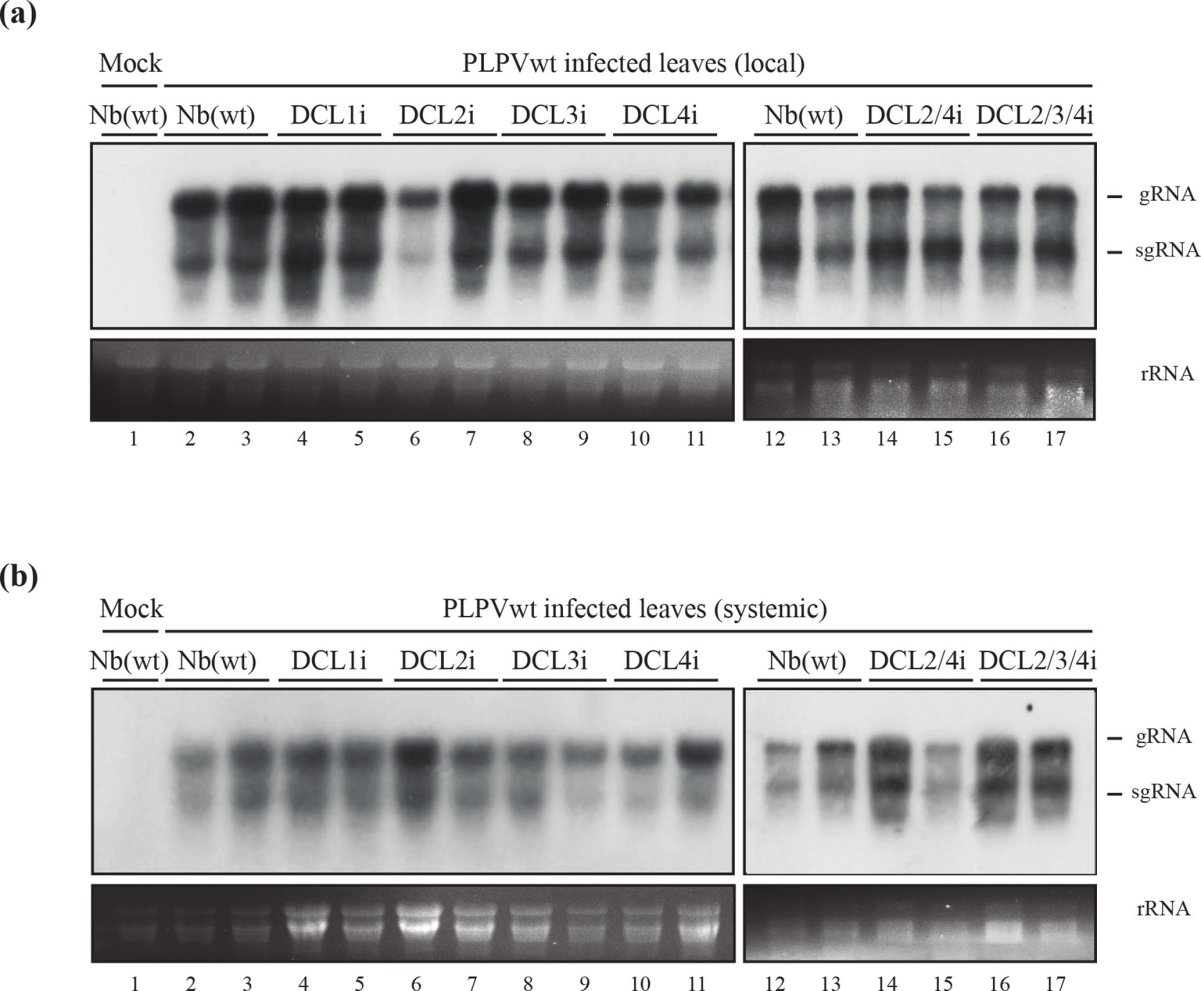
Accumulation of wt PLPV in lines of *N. benthamiana* with downregulated DCL content. The wt PLPV was agroinoculated in wt and in single (DCL1-4), double (DCL2/4) and triple (DCL2/3/4) RNAi lines. Northern blot analysis of local (a) and systemic (b) leaves collected at 7 days p.i. and 34 days p.i., respectively, was performed with a PLPV-specific riboprobe. Positions of the PLPV genomic (g) and subgenomic (sg) RNAs are indicated at the right of the blots and ethidium bromide staining of rRNAs is included below the blots as loading controls. Two distinct samples are shown for each virus–plant line combination. Plant batches for each experiment included four–six plants and experiments were repeated at least three times. Apparent differences in PLPV titers in systemic leaves of wt and DCLi lines correlate with distinct RNA loads and/or correspond to intra-experiment variations.

In order to check whether the effects of DCL downregulation were also apparent in an infection initiated with a VSR-competent virus, the same DCL RNAi lines were inoculated with the wt PLPV. As the control wt *N. benthamiana* plants, all inoculated DCL RNAi lines remain asymptomatic during their lifespan. Analysis of local and systemic leaves collected at 7 days p.i. and 34 days p.i., respectively, did not reveal significant differences in viral titers between DCL RNAi plants or when compared with wt *N. benthamiana* plants (Fig. 3), underlining the desirability of using a VSR-disabled virus when analysing participation of silencing factors in the host response against the infectious agent.

Collectively, the results of this section provide genetic evidence on the major role of DCL4 and, to lesser extent, DCL2 in anti-PLPV defence.

### AGO2 plays a key role in the race against PLPV in *N. benthamiana*

**Fig. 4.**
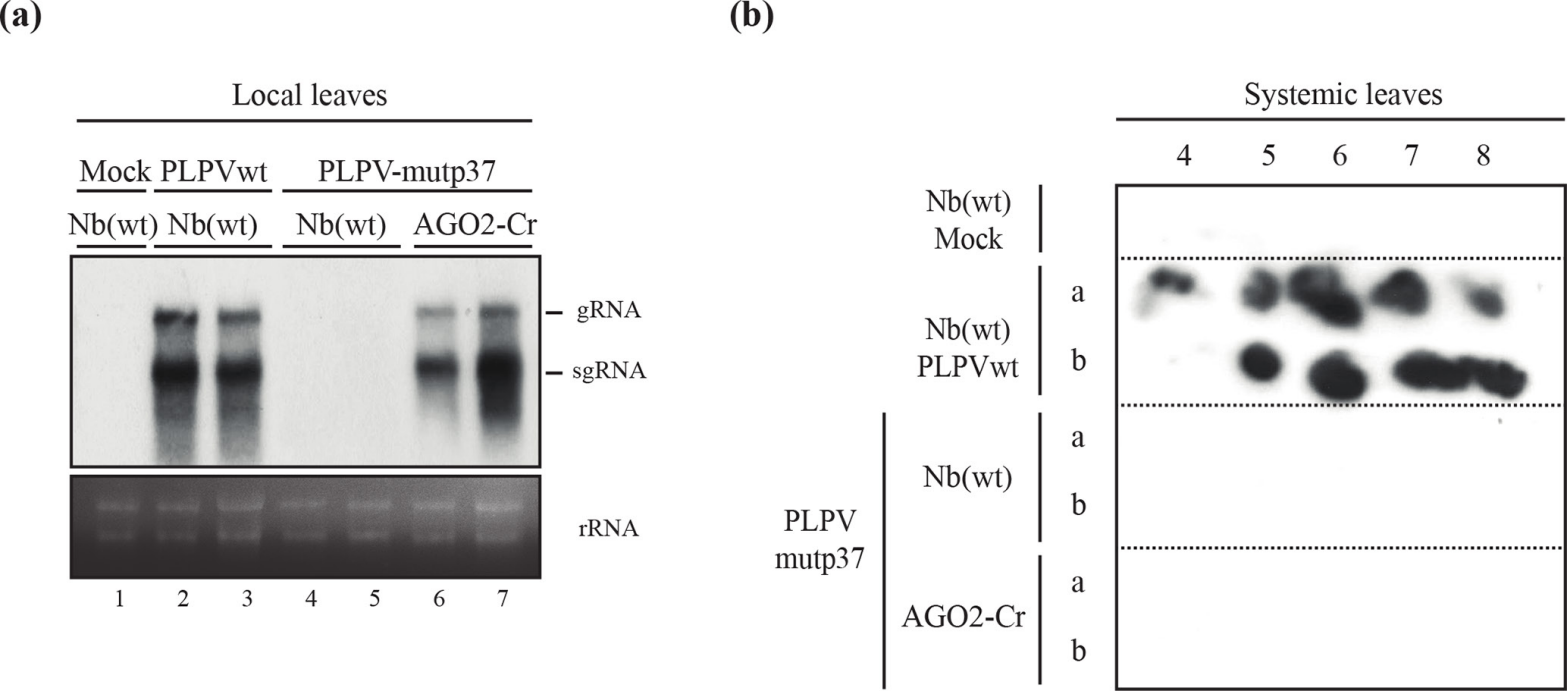
Accumulation of a VSR-deficient PLPV mutant in an AGO2-inactivated *N. benthamiana* line. PLPV-mutp37 was agroinoculated in wt *N. benthamiana* and in *N. benthamiana* AGO2-Cr line (with AGO2 inactivated through a CRISPR/Cas9 approach) and local and systemic leaves were harvested at 7 days p.i. and 42 days p.i., respectively, for further analysis. For comparison purposes, wt PLPV was inoculated in parallel on wt *N. benthamiana* plants and local and systemic leaves were collected at the same times. (a) Northern blot analysis of local leaves of different *N. benthamiana* plants (as shown above the lanes) inoculated with either wt PLPV (lanes 2–3) or PLPV-mutp37 (lanes 4–7). Leaves from mock-inoculated plants (lane 1) were used as negative controls. Hybridization was performed with a ^32^P-labelled PLPV-specific riboprobe for detection of PLPV genomic (g) and subgenomic (sg) RNAs whose positions are indicated at the right. Duplicate samples are shown for each virus–plant line combination. Ethidium bromide staining of rRNAs is included below the blots as loading controls. (b) Representative tissue printing hybridization of systemic leaves from mock and PLPV-inoculated plants using a PLPV-specific riboprobe as above. Results from two plants (a and b) are shown for every virus–plant line combination but the outcome was identical for other plants of each series. Plant batches for each experiment included four–six plants and experiments were repeated a minimum of three times.

The first definitive evidence on the involvement of an AGO protein in antiviral defence was obtained with AGO1 of *A. thaliana* [15]. However, later on, studies with both *A. thaliana* and *N. benthamiana* supported the participation of AGO2 in the defence against distinct types of viruses [14, 35, 41–45]. The generation of *N. benthamiana* lines in which AGO2 was inactivated through a CRISPR/Cas9 approach has been reported [35] and we decided to use one of these lines, hereafter AGO2-Cr, to evaluate the relevance of this slicer in anti-PLPV silencing. Hence, PLPV-mutp37 was inoculated onto *N. benthamiana* AGO2-Cr plants along with wt *N. benthamiana* plants and virus accumulation in local leaves was analysed at 7 days p.i. through a Northern blot assay. The results showed very low accumulation of the VSR-deficient virus in leaves of wt *N. benthamina*, as expected (Fig. 4a, lanes 4–5). However, the disabled virus reached high levels in leaves of *N. benthamiana* AGO2-Cr (Fig. 4a, lanes 6–7) that were indeed almost comparable with those usually detected for the wt virus in wt *N. benthamiana* (Fig. 4a, lanes 2–3). Despite the increment in the titers of PLPV-mutp37 in the local leaves of *N. benthamiana* AGO2-Cr, the virus could not be detected in systemic leaves at 42 days p.i. (Fig. 4b), indicating that inactivation of AGO2 is not sufficient to fully restore the infection capabilities of the VSR-deficient PLPV.

**Fig. 5.**
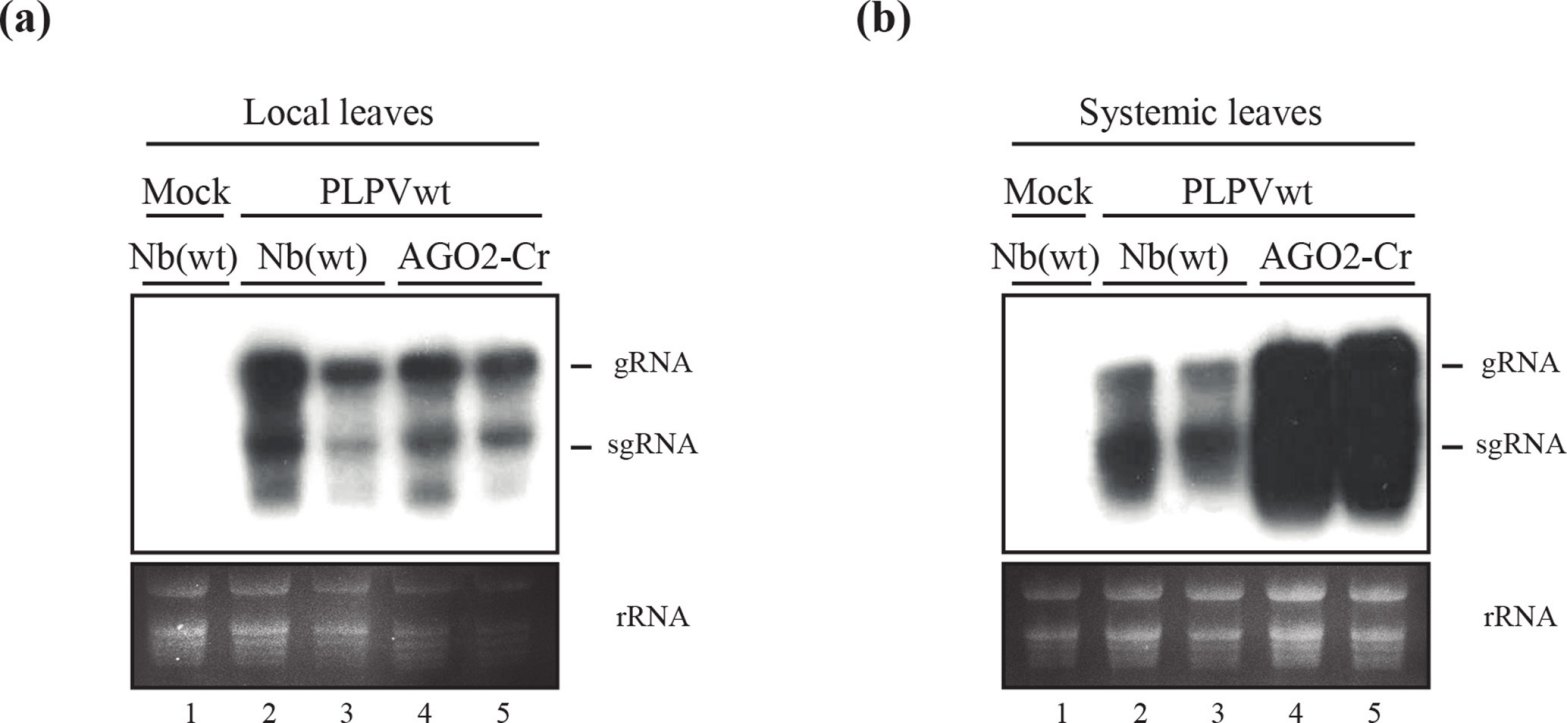
Accumulation of wt PLPV in an AGO2-inactivated *N. benthamiana* line. The wt PLPV was agroinoculated in wt *N. benthamiana* and in *N. benthamiana* AGO2-Cr line (with AGO2 inactivated through a CRISPR/Cas9 approach). Northern blot analysis of local (a) and systemic (b) leaves collected at 7 days p.i. and 42 days p.i., respectively, was performed with a PLPV-specific riboprobe. Positions of the PLPV genomic (g) and subgenomic (sg) RNAs are indicated at the right of the blots and ethidium bromide staining of rRNAs is included below the blots as loading controls. Two distinct samples are shown for each virus–plant line combination. Plant batches for each experiment included four–six plants and experiments were repeated at least three times.

Similarly to the experimental schedule followed in the previous section, we wanted to check whether the effects of AGO2 inactivation could also be appreciated in viral infections launched with wt PLPV. Northern blot analysis did not reveal significant differences in wt PLPV titers between local leaves of wt *N. benthamiana* and *N. benthamiana* AGO2-Cr plants (Fig. 5a). However, wt PLPV accumulation levels in systemic leaves of *N. benthamiana* AGO2-Cr plants were clearly above those detected in systemic leaves of wt *N. benthamiana* (Fig. 5b) and, thus, effects of AGO2 impairment were evident even with an entirely functional virus. As observed with control wt *N. benthamiana* plants, inoculated AGO2-Cr plants did not show any apparent symptom. Altogether, the obtained data unambiguously show that AGO2 plays a fundamental role in *N. benthamiana* anti-PLPV fight.

## Discussion

In this work, the importance of dicing and slicing activities in the *N. benthamiana* response against PLPV has been evaluated following a genetic approach. In a previous work, the high proportion of vsRNAs detected in PLPV-infected plants together with their RDR6-independent production as well as the balanced presence of plus and minus vsRNA strands, suggested that most of these sRNA species corresponded to primary vsRNAs efficiently produced from viral dsRNA replication intermediates [32]. Such efficient targeting of viral replication forms could be sufficient by itself to restrain infection, keeping viral titers at low levels and making the participation of slicing activities dispensable. However, through the employment of *N. benthamiana* lines with DCL or AGO2 functions impaired, here we have clearly shown that both types of activities, dicing and slicing, contribute to viral clearance.

The genetic evidence obtained on the relative degree of involvement of DCL4 and DCL2 in anti-PLPV defence is consistent with the distribution of vsRNA size classes in PLPV-infected *N. benthamiana* plants [32] and is also in agreement with data obtained in other plant-virus combinations [10, 11, 38, 39]. No clear evidence for involvement of DCL3 in the combat against PLPV was achieved though, from previous data, it could already be anticipated a very minor role for that enzyme [32].

The finding of the participation of AGO2 in anti-PLPV silencing extends over the number of viruses that are affected by this particular slicer. For some time, studies performed in *A. thaliana* pointed to AGO1 as the main effector in antiviral defence [15, 16, 46]. However, subsequent studies with such model plant species revealed that AGO2 also plays an important role in the plant race against viruses, especially, though not only, when facing viruses that are not known to target AGO1 [14, 41, 47–50]. Information about this topic in other plant species is, comparatively, much less known and essentially restricted to *Oryza sativa* and *N. benthamiana* [6]. In the latter case, evidence for the involvement of AGO1 in the response against tomato ringspot virus has been obtained [51]. In addition, *N. benthamiana* AGO2 has been reported to be involved in the fight against distinct types of viruses [35, 42–44, 52], suggesting that this specific AGO is a key antiviral factor in this plant species. However, more studies are needed to evaluate the relative importance of AGO1 and AGO2, or of other AGOs, in *N. benthamiana* antiviral silencing. As in other hosts, such importance is likely to be virus-dependent. This dependency may be influenced by the way of action of the corresponding VSR, by the expression of AGO(s) at the right place at the right time, by the accessibility of viral substrates [6] and, also, by the characteristics of the vsRNAs present in infected tissue. More specifically, the identity of the 5´-terminal nucleotide is known to determine, to great extent, sRNA loading in a particular AGO [53, 54]. Interestingly, AGO2 has preferred binding affinities by sRNAs with a 5´-terminal A and PLPV sRNAs with such terminal residue were the most abundant in infected plants (29.96 % [32]). Nevertheless, they were closely followed by vsRNAs with a 5´-terminal U (29.76 %) that are presumably loaded into AGO1 and, thus, this AGO (or even additional AGOs since non-negligible amounts of PLPV sRNAs with 5´-terminal C or G were found in infected tissue) could likewise be taking part in the contest against PLPV. It is also worth noting that PLPV p37 has been shown to interact with AGO1 [31] and, on the assumption that AGO1 function is effectively undermined by this interaction, AGO2 could be acting as a second defence layer against PLPV, as proposed in other instances [14]. In any case, the participation of other AGOs in addition to, or in cooperation with, AGO2 in the *N. benthamiana* struggle against PLPV may be critically assessed as plant lines with other inactivated slicers become available. Interestingly, CRISPR/Cas9-mediated inactivation of the two AGO1 homologues identified in *N. benthamiana* has been recently reported [55], opening the possibility for future evaluation of the involvement of these particular endoribonucleases in anti-PLPV defence.

In agreement with that observed in other plant–virus interactions (e.g. [15, 16, 35]), the effects of the impairment of silencing components on PLPV infection were particularly obvious when a VSR-defective virus was employed. Strikingly, such effects were also apparent in systemic leaves of *N. benthamiana* AGO2-Cr plants infected by the wt virus in contrast with that observed with the distinct DCL RNAi lines. Though these differences may be due to several reasons, a likely possibility is that, whereas inactivation of AGO2 through CRISPR/Cas9 is complete, downregulation of DCL factors through RNAi is only partial and, thus, the remaining DCL amounts may still contribute to limit infection. In addition, RNAi may be weakened by the PLPV VSR, raising DCL levels. The paradox of studying components of RNA silencing by using RNAi techniques has been already pointed out by some authors [35, 42], as this approach relies on those molecules that are intended to be knocked down. Hence, CRISPR/Cas9 approaches might be more suitable when analysing the role of RNA silencing components in antiviral defence. Nevertheless, both strategies, RNAi-mediated mRNA downregulation and CRISPR/Cas9-medited gene inactivation, may be employed as complementary approaches, being the first one particularly useful when the targeted gene is essential at specific developmental stages and, thus, gene knockout may result in lethality as reported, for instance, for DCL1 [56].

The lack of systemic infection by the PLPV-mutp37 in double/triple DCL RNAi or AGO2-Cr lines is puzzling as this virus mutant accumulates at considerably high levels in local leaves of the modified plants. The ultimate reasons of this observation are unclear but they could be related with the need to reach a defined threshold level for effective virus exit from the local leaves to the vascular system and/or a defined concentration of ‘circulating’ virus in the vascular system to efficiently enter systemic leaves [57]. In these scenarios, minor downward variations in virus titers may preclude any of these processes. In addition, the silencing components could be somehow involved in virus systemic movement, as has been suggested for DCL4 [58]. Another alternative that cannot be ruled out concerns the particular mutant virus that has been employed in this study. As indicated before, that mutant harbours a single aa replacement in p37 that abolishes its VSR function but preserves its encapsidation capability. Such mutation also impairs the nucleolar localization of the protein, which, according to our recent results, may be detrimental for virus accumulation [59]. We cannot discard that such nucleolar localization may have also an impact for virus long-distance movement and systemic infection, paralleling, saving the distances, that reported for the ORF3-encoded movement protein of umbraviruses [60].

Finally, there are some examples reported in which the impairment of silencing components led to an increment in wt virus accumulation that, even when being small and/or transient, had drastic consequences on viral symptoms [14, 35, 55]. Conversely, no phenotypic effects were observed in any of the engineered *N. benthamiana* lines infected by wt PLPV, despite the considerable increase in wt virus titers observed in systemic leaves of AGO2-Cr plants (Fig. 5b). This observation suggests that the virus has robustly evolved to pass unnoticed, likely because its asymptomatic condition favours its prevalence. Whether higher augmentations in wt PLPV titers may result in plant disease remains to be seen.
